# Gingival Bleeding in a Child with Fanconi Anemia: A Case Report and Literature Review

**DOI:** 10.1155/2020/3161053

**Published:** 2020-03-09

**Authors:** Dorsaf Touil, Rahma Bouhouch, Raoua Belkacem Chebil, Lamia Oualha, Nabiha Douki

**Affiliations:** University of Monastir, Dental Faculty of Monastir, Research Laboratory LR12ES11, Tunisia

## Abstract

Fanconi anemia (FA) is a rare autosomal recessive disorder characterized by multiple congenital abnormalities, bone marrow failure, and higher susceptibility to malignancies, especially to head and neck carcinomas. Only few reports about the oral manifestations of FA are available. The main reported oral conditions associated with FA are microdontia and advanced periodontitis. The aim of this paper was to report a case of a 10-year-old patient with FA presenting severe spontaneous gingival bleeding, as well as to discuss the role of the dentist in the management and treatment of this condition.

## 1. Introduction

Fanconi anemia (FA) is a genetic disorder occurring in 1-2 per 100000 births [1]. In 90% of the cases, it is caused by a mutation in 3 genes: FNCA, FANCC, and FANCA. All the genes implicated in the development of FA DNA are involved in the crosslink repair pathway. Thus, patients with FA exhibit impaired capacity to repair the DNA crosslinks [2].

Clinically, FA is often characterized by multiple congenital abnormalities, progressive bone marrow failure, and an increased risk of developing squamous cell carcinomas, particularly head and neck squamous cell cancer, at a young age [3]. The clinical course of FA begins in the first decade of life, during which patients exhibit congenital abnormalities, hematological disturbances, or both [4]. Their physical abnormalities may include a short stature, abnormal skin pigmentation, thumb and radial anomalies, structural renal defects, microcephaly, and delayed development that are recognizable at birth and/or during early childhood [5].

There are only few reports with regard to the oral manifestations of FA. The main described oral conditions associated with FA are microdontia, missing teeth, and advanced periodontitis [6].

Patients with severe periodontitis may present gingival bleeding, tooth mobility, and halitosis. These manifestations are often related to the host defense impairment seen in these subjects, which increases their susceptibility to periodontal infection, and to the poor oral hygiene [6, 7].

The thrombocytopenia exacerbates the gingival bleeding and complicates its management, especially in young patients who are affected not only by the disease but also by the heavy treatment that often requires long hospitalization periods, which dramatically reduces their access to oral hygiene [8].

The aim of this paper was to report a case of a 10-year-old patient with FA presenting severe spontaneous gingival bleeding, as well as to discuss the role of the dentist in the management and the treatment of this condition.

## 2. Case Report

Written informed consent of the patient's father was obtained prior to this paper's publication.

**Figure 1:**
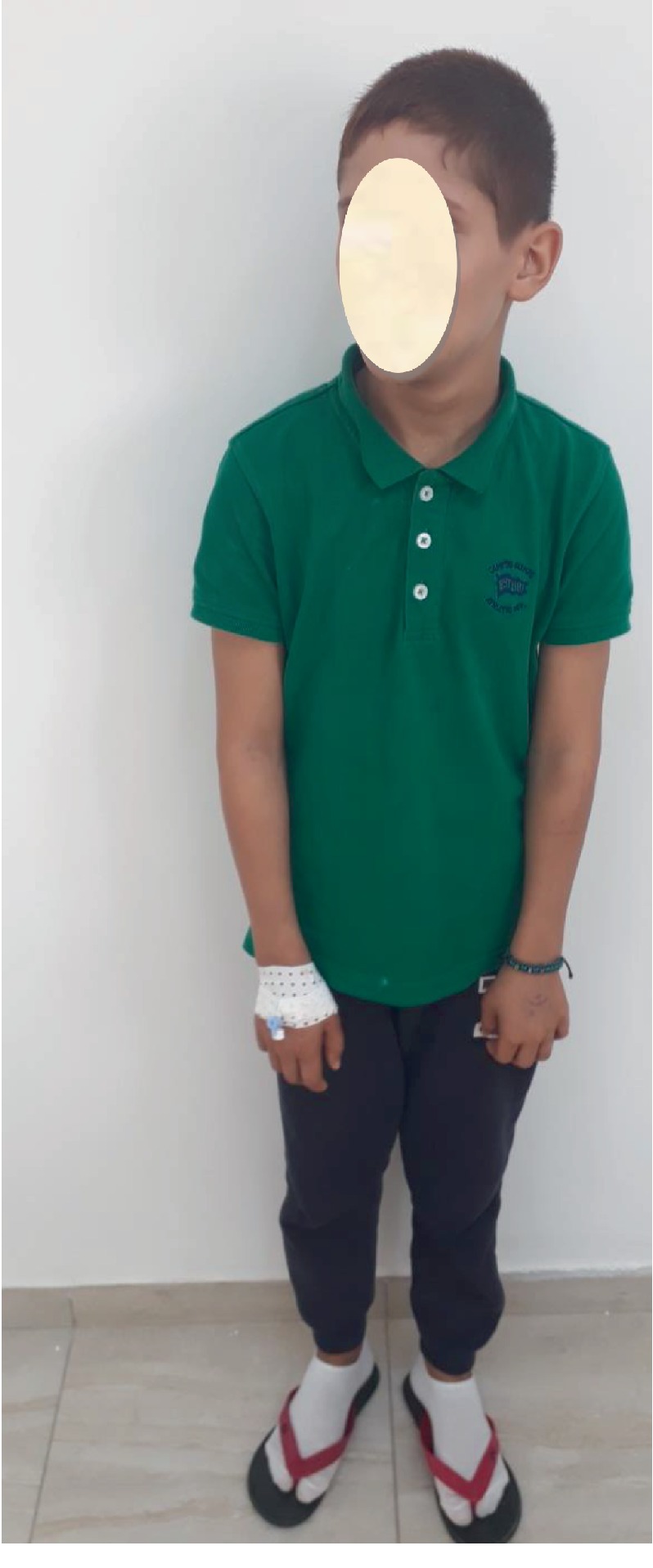
Physical examination of the patient showing a short stature.

A 10-year-old male patient (Figure 1) was referred by his hematologist to the Department of Oral Medicine at the University Hospital Sahloul, Tunisia, for spontaneous severe gingival bleeding during the last two days before consultation.

**Figure 2:**
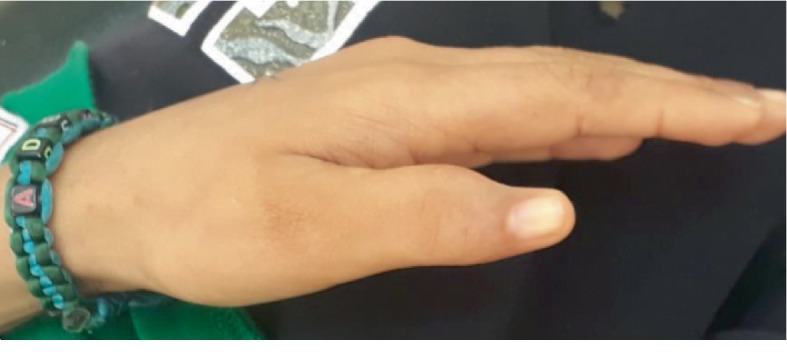
Operated bifid thumb.

The patient was diagnosed with FA at the age of five. The patient's family history was significant as he was the second child of a consanguineous marriage. His parents and sibling were healthy. The patient was operated for bifid thumbs at the age of 2 years (Figure 2) and for cryptorchidism at the age of seven.

The child's physical examination revealed a short stature (Figure 1). He had a pale skin as well as skin pigmentations in the form of “café au lait” spots that were noticeable on his face and arms.

The intraoral examination revealed petechial hemorrhages with oozing blood from the gingiva which was more evident in the lower anterior region with a blood collection in the lower vestibule.

His total blood count showed hemoglobin (Hb) = 6.2 g/dL, platelet (PLT) = 6000/*μ*L, and white blood cell (WBC) = 1170/mm^3^.

Given the patient's agitation, the pancytopenia, and the severe bleeding, no dental therapeutics were possible.

Our therapeutic approach consisted in the application of a sterile compress soaked in tranexamic acid (Exacyl 1 mL/10 L) in local compression for 5 minutes in each bleeding area. A prescription of tranexamic acid, four times a day, as a passive mouthwash was recommended. The patient was asked to avoid hot foods and drinks.

The patient was hospitalized to keep him under supervision and to correct his blood count. Given the FA-related severe thrombocytopenia, a platelet concentrate was necessary to raise the platelet count above 50000/*μ*L. The patient was therefore given a single unit of leuko-depleted and irradiated apheresis platelet.

We received the patient two days later—during his hospital setting—for dental scaling. His blood count revealed Hb = 8.2 g/L, PLT = 51000/mm^3^, and WBC = 1170/mm^3^.

**Figure 3:**
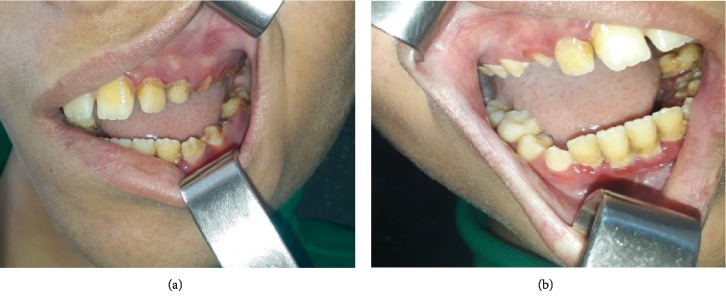
Oral examination showing poor oral hygiene and discolored teeth.

The oral examination showed poor oral hygiene and discolored teeth. However, the gingival bleeding was scarce (Figures 3(a) and 3(b)).

Sixty minutes before the dental scaling, the patient received 1.5 g ampicillin per os to reduce the risk of postoperative infection. We started by rinsing the patient's mouth for 2 minutes with 0.2% chlorhexidine solution; after that, manual supragingival scaling was performed under local anesthesia. The gingival bleeding was controlled by local compression using gauze saturated with tranexamic acid.

At the end of the treatment session, the patient and his parents received succinct instructions with regard to the importance of maintaining good oral hygiene, as well as the regular monitoring of his mouth.

The patient's parents were informed of the risks of developing malignancies, and they were asked to perform a regular mouth inspection to their child to detect any possible suspicious lesion. Follow-up sessions were scheduled.

## 3. Discussion

Fanconi anemia is a rare autosomal recessive syndrome caused by mutations in one of at least 19 genes. The proteins encoded by these genes act together to protect the genomic integrity [1].

FA belongs to a group of hereditary disorders clinically characterized by bone marrow failure, congenital malformations, and susceptibility to cancer [4].

The early symptoms of FA include multiple anomalies, such as skin pigmentation in the form of “café au lait” spots, short stature, thumb and genital anomalies, microcephaly, and cognitive impairment [9].

However, some patients with FA may not have any physical anomalies or skin lesions [10]. In the International Fanconi Anemia Registry (IFAR), a third of patients with FA were reported as not having any congenital malformations [11]. In our case, the patient was operated for both bifid thumb and cryptorchidism.

Due to its highly variable phenotype, diagnosis may be difficult in some patients. For this reason, tests such as chromosome breakage analysis and comet assays are often performed to reach a definitive diagnosis [4].

The most frequent hematological complications of FA are pancytopenia, anemia, thrombocytopenia, and leukopenia. Because of these complications, patients affected by FA may develop severe bleeding as well as recurrent infections [12]. At the time of consultation, our patient had pancytopenia, especially a severe thrombocytopenia, which may explain the severity of the gingival bleeding.

The oral manifestations are frequently mentioned in the clinical descriptions of FA, but their prevalence has not been well documented [6]. As reported in the literature, the main oral findings associated with FA are gingivitis (41.5%) and severe periodontitis (22.3%) [7]. The high predisposition of patients with FA to periodontal disease may be due to anemia and leukopenia [13]. Moreover, the medications applied during the immunosuppressive treatment, such as corticosteroids, may further reduce the immunological defense, thus leading to a higher risk for periodontal disease [7]. It is worth mentioning that the poor oral hygiene remains a major cause of periodontal disease. Our patient had very poor oral hygiene and was not motivated to take care of his dentition. The long hospitalization periods were found to be the reason behind his demotivation and negligence.

Oral petechial hemorrhages and spontaneous gingival bleeding are common in patients with FA and are related to the very low platelet counts [14]. In previous case reports, oral traumatic and petechial hemorrhage lesions were seen in 30% of the patients and they were associated with decreased platelet level [6].

In another study, gingival bleeding was noted in 16% of patients with aplastic anemia [14].

Aphthous lesions were also reported in the literature. Some authors associated recurrent aphthous ulcers with the hematological condition, particularly anemia and neutropenia [14].

Dental anomalies such as agenesis, supernumerary teeth, microdontia, rotation, transposition, delayed eruption, and discolored teeth have been identified [9]. Oyaizu et al. reported a case of a patient with FA having macroglossia and generalized microdontia [12]. In the study of de Araujo et al., 9.1% of the patients showed anomalies in teeth on panoramic radiographs [6]. In the case we report, the patient had a complete dental formula but all his teeth were discolored.

Few studies have cited the prevalence of dental caries in the FA population. Caries were found to be associated with poor oral hygiene and accumulation of plaque but not with the systemic condition [9]. A preventive dental treatment plan should be implemented for all FA patients. It should include motivation to strict oral hygiene, conservative treatment of decayed teeth, and sealing of pits and fissures of the occlusal surfaces.

A close cooperation between the hematologist and the dentist is necessary to prevent excessive hemorrhage during dental procedures, especially when performing invasive ones, such as dental extractions, subgingival scaling, block anesthesia, or oral surgery. The platelet count should then be raised to at least 50000 *μ*L [12, 15]. Thus, the dental treatment for these patients should be pursued in a hospital setting to ensure an appropriate follow-up of any acute complications that may arise [14]. Our patient was hospitalized to be kept under close supervision. He was given a single unit of leuko-depleted and irradiated apheresis platelets to raise his blood count. His platelet count was raised to 51000/mm^3^, which was imperative to perform dental scaling without any excessive bleeding.

Moreover, dentists must use local hemostatic measures following the dental extraction in these patients as well as sutures and topic agents, such as oxycellulose, gelatine, fibrin glue, and cyanoacrylate [16, 17]. An antifibrinolytic treatment using tranexamic acid (TA) is also recommended to prevent postoperative bleeding. Various reports have described the successful local use of TA for the reduction of the oral bleeding [18]. In the case we report, a successful control of the gingival bleeding was obtained using compressive gauze saturated with TA.

Patients with FA must receive antibiotics if the absolute neutrophil count is less than 500/*μ*L. There is no consensus on antibiotic prophylaxis for a neutrophil count greater than 500/mm^3^. Antibiotic prophylaxis, typically with amoxicillin or clindamycin, should be given one hour before invasive dental procedures and should be continued until mucosal healing to prevent serious complications [19]. Dentists should also consider prescribing an antiseptic mouthwash prior to the dental management. The use of chlorhexidine mouth rinse, 30 min before the dental procedure, may be effective in reducing the risk of postoperative infection, as it has been shown to be effective in reducing plaque and gingival inflammation [12]. As antibioprophylaxis, our patient received 1.5 g ampicillin per os, 60 min before the procedure. We also used a chlorhexidine mouthwash few minutes before the gingival scaling. Our patient did not have any local or systemic complications.

The propensity to develop cancer is well documented in patients with FA. This condition is associated with a 30- to 80-fold increased risk of developing solid tumors [20] which is thought to be related to the chromosomal instability [1, 3].

The squamous cell carcinomas of the head and neck (HNSCC) represent the most frequent solid tumors. Patients with FA have up to 500-fold increased risk of developing such tumors compared to the general population [2], and these tumors are commonly located in the oral cavity [21]. Moreover, the International Fanconi Anemia Registry study observed a 20% higher risk of SCC in FA patients aged over 40 years. The median age of HNSCC diagnosis was 32 years compared to a median age of 63 in the general population [1]. On the other hand, HNSCC in the FA population have a poor prognosis and are known for their aggressive growth capacity and propensity to metastasize [5, 22].

Tobacco smoking and alcohol consumption, which are common risk factors for HNSCC in the general population, do not seem to be significant risk factors in FA patients [1, 17]. However, some authors have suggested that HPV is a leading risk factor for SCC of the head and neck in this population where 84% of the SCC contained quantifiable HPV DNA [1, 3].

Patients who underwent bone marrow transplantation have a greater incidence of malignancy development [3]. A recent study reported a 4.4-fold higher risk for SCC in FA patients who received transplants [23]. According to the authors, chronic GVHD seem to be a strong risk factor for SCC. In addition, it is suggested that in these patients, the disease onset also occurs approximately 10 years earlier [3].

Because of the high risk of developing oral cancer, an early screening together with a careful examination of the mucosal tissues is very important. Specialists recommend their patients to have an oral examination at 3- to 6-month intervals [24]. These sessions should include instruction in maintaining excellent oral hygiene. Self-examination of the mouth is recommended as a secondary preventive measure [25]. Even if our patient did not have any suspicious oral lesions, his parents were informed of the higher risk of developing carcinomas and were therefore taught how to perform meticulous examination to their child's oral cavity.

To conclude, dealing with the severe gingival bleeding of our young patient required the correction of his blood count and the use of an antifibrinolytic agent in local compression and a mouthwash. All these measures were necessary before starting the local treatment which consisted in a supragingival scaling that was aimed at reducing the local inflammation. These measures were efficient for a successful control of the severe gingival bleeding.

Still, no consensus is available with regard to the dental treatment modalities of patients with Fanconi anemia.
